# In Silico Exploration of *Mycobacterium tuberculosis* Metabolic Networks Shows Host-Associated Convergent Fluxomic Phenotypes

**DOI:** 10.3390/biom12030376

**Published:** 2022-02-28

**Authors:** Guillem Santamaria, Paula Ruiz-Rodriguez, Chantal Renau-Mínguez, Francisco R. Pinto, Mireia Coscollá

**Affiliations:** 1 I^2^SysBio, University of Valencia-FISABIO Joint Unit, 46980 Paterna, Spain; gsantamaria@fc.ul.pt (G.S.); paula.ruiz-rodriguez@uv.es (P.R.-R.); chantal.renau@uv.es (C.R.-M.); 2 BioISI—Biosciences & Integrative Sciences Institute, Faculty of Sciences, University of Lisboa, 1749-016 Lisboa, Portugal

**Keywords:** *Mycobacterium tuberculosis*, lineage, host association, genome-scale metabolic model, metabolic networks

## Abstract

*Mycobacterium tuberculosis*, the causative agent of tuberculosis, is composed of several lineages characterized by a genome identity higher than 99%. Although the majority of the lineages are associated with humans, at least four lineages are adapted to other mammals, including different *M. tuberculosis* ecotypes. Host specificity is associated with higher virulence in its preferred host in ecotypes such as *M. bovis*. Deciphering what determines the preference of the host can reveal host-specific virulence patterns. However, it is not clear which genomic determinants might be influencing host specificity. In this study, we apply a combination of unsupervised and supervised classification methods on genomic data of ~27,000 *M. tuberculosis* clinical isolates to decipher host-specific genomic determinants. Host-specific genomic signatures are scarce beyond known lineage-specific mutations. Therefore, we integrated lineage-specific mutations into the iEK1011 2.0 genome-scale metabolic model to obtain lineage-specific versions of it. Flux distributions sampled from the solution spaces of these models can be accurately separated according to host association. This separation correlated with differences in cell wall processes, lipid, amino acid and carbon metabolic subsystems. These differences were observable when more than 95% of the samples had a specific growth rate significantly lower than the maximum achievable by the models. This suggests that these differences might manifest at low growth rate settings, such as the restrictive conditions *M. tuberculosis* suffers during macrophage infection.

## 1. Introduction

*Mycobacterium tuberculosis* is the causative agent of tuberculosis disease (TB), which affects a wide range of mammals, including humans. Tuberculosis is the second cause of human infection-related death, right after COVID-19 [1]. The ability to produce disease in livestock also makes *M. tuberculosis* a major concern from an economic point of view [2].

*M. tuberculosis* is composed of several phylogenetic lineages that can be broadly divided into animal- and human-associated, according to the host they are commonly isolated from. Human-associated *M. tuberculosis* lineages comprise L1 to L4 and L7, commonly referred to as *Mycobacterium tuberculosis sensu stricto*, L5 and L6, traditionally known as *M. africanum*, and recently described L8 and L9 [3,4,5,6,7]. On the other hand, there are four animal-associated lineages that compose several ecotypes with specific host associations: A1, A2, A3 and A4 [8,9]. A1 to A3 infect primarily wild animals, whereas the members of A4 are mainly isolated from cattle and goats and are referred to as *M. bovis* or *M. caprae*, respectively [8,10]. Host specificity in *M. bovis* originates host-dependent virulence, producing a mild version of tuberculosis disease in humans with human-to-human transmission being extremely rare [11]. Conversely, *M. tuberculosis* is attenuated in cattle [12,13]. It is unclear if this phenotype can be extended to other *M. tuberculosis* lineages or hosts, and which molecular mechanisms are involved.

*M. tuberculosis* lineages have a high genomic identity (>99%), product of its clonal evolution and absence of horizontal gene transfer [10,14]. Despite this similarity, they show a wide range of pathogenicity-related phenotypes, including host association [15,16]. L5, L6 and L9 are of particular interest in the study of host association from a phylogenetic point of view, because they are the human-associated lineages that are closer to the animal-associated ones [17]. Therefore, the study of the differences between L5, L6 and L9 and the animal-associated lineages could shed light on the mechanisms of adaptation to different hosts. However, the high genomic identity between *M. tuberculosis* lineages hinders the identification of genomic signatures correlated to host adaptation phenotypes. The study of metabolism, which is functionally closer to the phenotype, could reveal details about *M. tuberculosis* infections and host association phenotypes. However, obtaining of metabolomic or lipidomic data in the context of infection can be challenging. A suitable and alternative approach is the use of genome-scale metabolic models (GEMs).

GEMs are metabolic networks built from genome annotations, which are used to infer the possible reactions within the organism. After the application of constraints based on stoichiometry and experimental data, the model can be used to predict metabolic phenotypes in a determined medium composition [18]. Flux Balance Analysis (FBA) is typically used for this purpose: steady state is assumed under an objective function, commonly set to maximize growth rate [19]. This approach might not be optimal to study *M. tuberculosis* infection, as the hostile environment within the macrophages’ phagosomes restricts bacterial growth [20]. Furthermore, growth is extremely limited during dormancy. Therefore, metabolic strategies involved in *M. tuberculosis*’ virulence may be unrelated to growth maximization. An alternative approach is the use of flux sampling, where the solution space of the metabolic model is explored by random sampling without assuming any objective reaction to be maximized [21]. 

GEMs have been used with success to predict phenotypes such as substrate utilization, gene essentiality, production of virulence factors or response to oxidative stress in different microorganisms [22,23,24]. In the context of *M. tuberculosis,* not only have they been used with similar aims [25,26], but also to determine the metabolic rewiring produced by subinhibitory concentrations of antibiotics, to assess the effect of SNPs in metabolic genes or to determine condition-specific biomass compositions, such as during infection [27,28,29]. The metabolic flux diverting from several pathways as a stress response has also been experimentally validated [25]. However, most of these studies focus on the laboratory strain H37Rv, except for the work of Øyås et al., which covers from L1 to L7 [29]. Studies analyzing how genomic differences between animal and human *M. tuberculosis* lineages could impact their fluxomic phenotypes are still lacking.

In this study, we analyzed the genomes of ~27,000 clinical isolates spanning all the known lineages of *M. tuberculosis* with a combination of unsupervised and supervised methods. We determined which genomic signatures contribute more strongly to the separation of animal- versus human-associated lineages. However, most of the signatures were not exclusive of either human- or animal-associated lineages. We therefore adapted a previously available *M. tuberculosis* GEM, iEK1011 2.0 [26,30], to model each one of the *M. tuberculosis* lineages. With the obtained models we predicted how the genomic differences might translate into metabolic phenotypes. We observed that genomic differences apparently uncorrelated to host specificity introduce perturbations that propagate through the metabolic network, producing convergent metabolic phenotypes correlated with host association and possibly involved in host adaptation.

## 2. Results

### 2.1. M. tuberculosis Polymorphisms Allow to Partially Separate Isolates According to Host Association with Unsupervised Methods

To assess to what extent single nucleotide polymorphisms (SNPs) have a role in host specificity, we retrieved the available Illumina genomes of *M. tuberculosis*: ~27,000 clinical isolates spanning all the known lineages of the *M. tuberculosis* (L1, L2, L3, L4, L5, L6, L7, L8, L9, A1, A2, A3 and A4) (Supplementary Table S1). We built a set of potentially deleterious SNPs including SNPs causing premature stop codons and non-synonymous SNPs potentially affecting protein function (PROVEAN score ≤ −2.5) [31]. A correspondence analysis of these data shows that L7 and L8 are in the periphery of the bidimensional space (Supplementary Figure S1). These two lineages are the ones with the longest phylogenetic branches. L8, furthermore, is the most basal of the lineages [6]. This early separation in phylogeny along with the branch length might explain the differential accumulation of SNPs that separates these two lineages from the rest in the correspondence analysis. The remaining lineages aggregate together. However, we can still detect that L6, L9 and the animal-associated lineages are separated from the human lineages L1 to L5. This result partially mimics phylogeny, where L5, L6, L9 and all animal-associated lineages form a monophyletic group [8].

**Figure 1:**
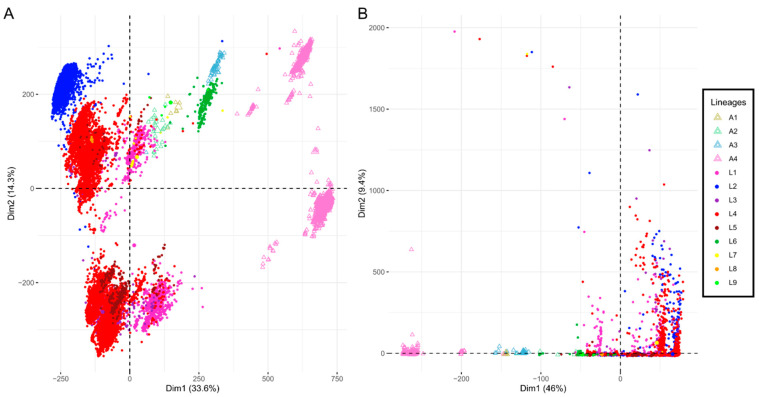
Principal Component Analysis of ORF deletion percentage. (**A**). All ORFs. (**B**). ORFs annotated as enzyme-coding. Each point represents a genome, which is colored according to the lineage it belongs to.

In parallel, we determined the percentage of deletion of each ORF, using the read depth per genomic position, and analyzed it using Principal Component Analysis (PCA). The two major components show a clear separation between the isolates belonging to either animal- or human-associated lineages (Figure 1A). L6 and L9 are close to A1, A2 and A3, which are the animal lineages that are closer to the human-associated ones in phylogeny. As mentioned before, L6 and L9 emerge from a common ancestor of the animal-associated clades. We are therefore obtaining an approximation of the *M. tuberculosis* phylogeny by using only genomic deletions. This separation between animal- and human-associated lineages becomes more evident when using only enzymatic gene deletions (Figure 1B). In this case, L6 and L9 are still close to the animal lineages, but with less overlap. This difference in enzymatic content suggests that animal- and human-associated lineages might have distinct metabolic networks. The resulting metabolic profiles might have a role in host adaptation.

### 2.2. Supervised Methods Do Not Identify Enough Genomic Signatures Correlated to Host Association to Explain the Phenotype

**Figure 2:**
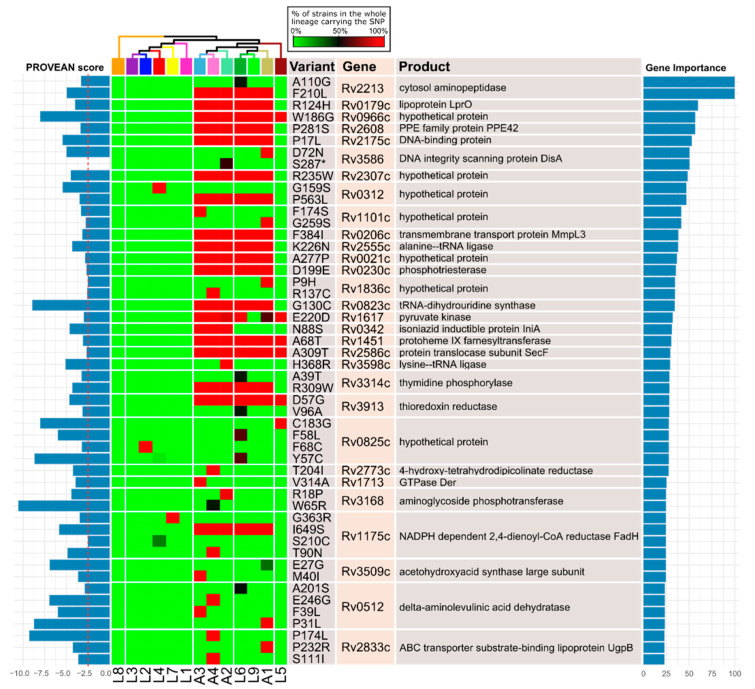
Supervised analysis of potentially deleterious SNPs. Potentially deleterious SNPs included amino acid substitutions predicted by PROVEAN to be deleterious (leftmost bar plot, score ≤ −2.5, indicated in dashed red line) and premature stop codon introducing SNPs. Top 25 genes were sorted according to their importance in the classification (rightmost bar plot). The phylogenetic relationship between the lineages is shown at the top.

We then used a supervised approach to determine what are the potentially deleterious SNPs and gene deletions that are producing the separation of animal- and human-associated isolates. Regarding the SNPs, we randomly selected a set of strains from each one of *M. tuberculosis* lineages and trained a random forest classifier to determine what SNPs are more strongly correlated with either animal- or human-associated lineages, keeping the top 25 most important genes in the classification (Figure 2). The model correctly classified all strains according to their host association (with 10-fold cross-validation). We could not find a set of genes with potentially deleterious SNPs restricted to either animal- or human-associated lineages. The most informative SNPs are present in all animal lineages, but also in L6 and L9, meaning that they probably appeared just once in the common ancestor of these lineages. There are also a couple of genes with potentially deleterious mutations restricted only to animal-associated lineages, but not in the four of them. One is *iniA* (Rv0342), which codes for an efflux pump involved in isoniazid and ethambutol tolerance and its expression is induced after treatment with isoniazid [32,33]. This gene has a single mutation in lineages A2, A3 and A4, probably appearing in their common ancestor in a single event. The other is Rv0512 (*hemB*), which codes for delta-aminolevulinic acid dehydratase, involved in the synthesis of cobalamin, a cofactor of many enzymes [34]. This gene has three different non-synonymous SNPs restricted to three out of four animal-associated lineages (Figure 2). These SNPs might produce differences in virulence or lipid metabolism. Most of the informative genes have potentially deleterious SNPs in the animal lineages but also in L6 and L9, and some in L5. This suggests that these genes might not actually be related to host specificity.

**Figure 3:**
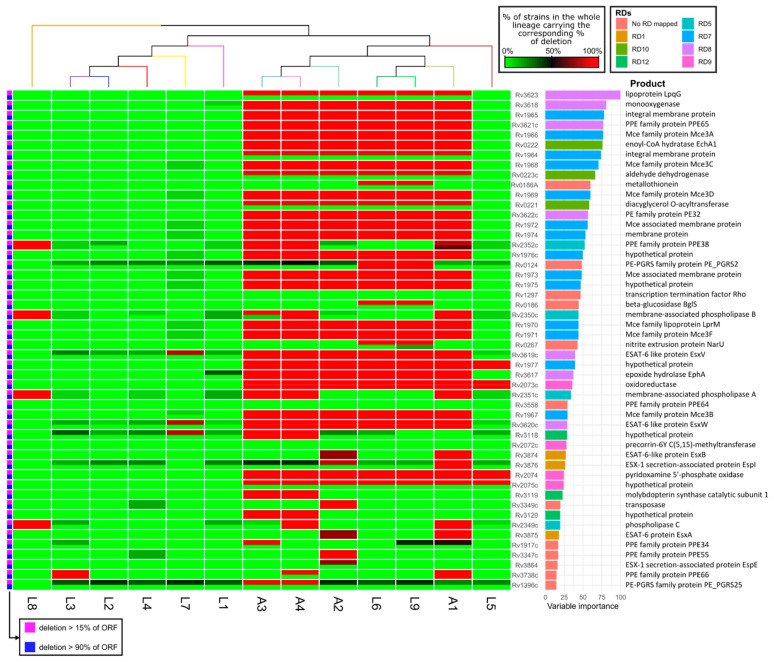
Supervised analysis of the deletion data. Random forest of the percentage of each ORF from the reference genome that was lost in each lineage, classifying the genomes as belonging to animal- or human-associated lineages. The variables (genes) are sorted according to their importance in the classification (rightmost barplot), and are colored depending on the RD they are located in. RDs are named according to Brosch et al. 2002. The heatmap corresponds to the percentage of isolates with a percentage of deletion higher of the 15% or 90% (magenta or blue, respectively, indicated in leftmost colorbar).

We also fitted a random forest model to deletion data, obtaining an accuracy higher than 0.99. The most important genes in the classification fall within several *M. tuberculosis* well-characterized long sequence polymorphisms known as regions of difference (RDs). In particular, the RDs that are important in the classification are RD1, RD5, RD7, RD8, RD9, RD10 (Figure 3) lost at different stages after the split of L5, L6, L9 and the animal lineages from the rest [8,17]. This implies that most of these genes are absent in the animal-associated lineages, but also in L5, L6 and L9. Despite the fact that some of the deleted genes in *M. africanum* and animal lineages might have some role in *M. tuberculosis* host association, they probably are not determinant, as they are not exclusive to a particular host. However, there are some exceptions. First, *esxA* and *esxB* (Rv3874 and Rv3875, respectively), which are part of RD1, have been lost independently in A1 and A2. RD1 is the main genetic modification involved in the attenuation of the BCG vaccine strain [35,36]. In the work by Brites et al., it was reported that these genes have been deleted at least four times in vivo: three times in A1, and once in A2 [8]. These recurrent deletions might be related with host and/or virulence adaptation, presumably linked to the jump from humans to animals. Rv3876, an ESX-1-associated protein which is also part of RD1, also appears in the rank. This gene, unlike *esxA* and *esxB*, is only completely deleted in A1 (as well as in *M. bovis* BCG). A2 has a partial deletion of Rv3876 in 77.5% of the isolates. Between 15% and 50% of the members of the remaining lineages carry a partial deletion in this ORF, with the exception of L7 and L8. L8 has this gene intact in all its members, whereas 7% of L7 isolates have lost 15 to 90% of this ORF. The other deletions showing a pattern of host association are part of RD5, which spans from Rv2349c to Rv2353c. Genes in RD5 might have been deleted at least four times independently, and it is the only marker deleted almost completely in all animal lineages and not in human lineages (except for L8) [8]. Rv2349c-Rv2351c code for PlcC, PlcB and PlcA, respectively, three phospholipases C linked to *M. tuberculosis* virulence and to the obtention of phosphate in the first stages of infection in the depleted environment of the phagosome [37,38]. Rv2352c and Rv2353c code for PPE38 and PPE39. PPE38 has been related to secretion of antigenic proteins, producing an increase in virulence in *M. tuberculosis* when mutated [39,40]. A big proportion of the isolates belonging to lineages A1 and L8 has the 5 genes completely deleted. A4 lost the three *plc* genes, *ppe38* and a fragment of *ppe39*, whereas A3 lost *plcA*, a fragment of *plcB, ppe38* and *ppe39*, whereas keeping *plcC* intact. This indicates that these deletions occurred at least 4 times along the phylogeny, concentrating mostly in animal clades [8,40]. The fact that L5, L6 and L9, despite being closely related to animal-associated lineages, have these genes intact supports its possible involvement in host specificity [8].

### 2.3. Modeling M. tuberculosis Lineages Suggest Possible Alternative Metabolic Pathways

We observed genomic differences between animal- and human-associated lineages in terms of enzymatic gene deletion and potentially deleterious SNPs. However, we could not pinpoint a set of genes whose presence was sufficient to explain host association. Consequently, we used a GEM to explore how these genomic signatures might translate into metabolic differences. We chose iEK1011 2.0 [26,30] as the base reconstruction for building 12 lineage-specific GEMs, as it is the most complete reconstruction at today’s date and because the genetic diversity of the genes included in this reconstruction supported the separation between human- and animal-associated strains using an unsupervised approach (Supplementary Figure S2, genes included in the model shown in Supplementary Table S2). iEK1011 2.0 models the H37Rv reference strain, an L4 strain, thus we used it as the model of this lineage. To infer the models for the remaining lineages we first deleted the genes that were absent in most of the genomes of the target lineage (see methods for details). Among the 12 models obtained (one per lineage, except L4), 2 of them had no viable steady state flux distribution: flux of biomass reaction was zero when running FBA in conditions simulating Middlebrock 7H9 OADC medium. We used these conditions to identify the removed genes whose absence was blocking growth, as Middlebrock 7H9 OADC is a medium commonly used in laboratory culture of mycobacteria and the isolates of all *M. tuberculosis* lineages can grow there. The growth blockage was caused by individual gene deletions.

Deletion of Rv1525 blocks growth of iEK1011 2.0 model. This gene has more than 90% of its sequence missing in 98.87% of L1 clinical isolates. Additionally, it appears to be completely deleted in a closed PacBio genome of this lineage (Accession Number AP018033.1) [41]. Rv1525 codes for a putative rhamnosyl transferase (*wbbL2*). This enzyme catalyzes the first step in arabinogalactan synthesis, a component of *M. tuberculosis* cell wall [42]. The last step of this pathway, three reactions downstream WbbL2, is the target of the antimycobacterial drug ethambutol [43]. All the genes downstream Rv1525 are essential in the H37Rv strain, both in rich and minimal medium, whereas Rv1525 is only essential in minimal medium [44,45,46,47]. This gene has been reported to be lost in some clinical isolates [48]. It is not clear if its absence produces a different biomass composition in these lineages or if there are other enzymes compensating for this mutation in L1 strains. In the model, the rhamnosyl transferase reaction requires two genes to be active: *wbbL2* and *wbbL1*. It might be possible that *wbbL1* (Rv3265c) is sufficient for catalyzing the reaction in vivo/in vitro. Indeed, this gene is essential for H37Rv in vitro [44,45,46,47].

L8 model was also not able to grow. The growth-blocking gene was Rv3281. In the available PacBio genome of L8 lineage (Accession Number CP048071.1) this gene appears shorter than the reference due to an internal deletion [6]. Rv3281 codes for the epsilon chain of bifunctional acetyl-/propionyl-coenzyme A carboxylase AccE5 [49]. This enzyme catalyzes the carboxylation of propionyl-CoA or acetyl-CoA into malonyl-CoA or methylmalonyl-CoA, respectively, in a biotin-dependent process [50]. Methylmalonyl-CoA can be used as a building block to synthesize the methyl-branched lipids that form *M. tuberculosis* cell wall, many of them involved in virulence [51,52]. The only two isolates of lineage L8 have a deletion in Rv3281, affecting 25.47% of its sequence length with respect to H37Rv’s ORF. This gene is essential for in vitro growth of H37Rv in several conditions [44,45,53]. Therefore, it is likely that other enzymes present in L8 but not accounted in the model are catalyzing this carboxylation. This might or not affect the lipidic composition of the isolates belonging to the L8 lineage.

We decided to keep these reactions instead of changing the model’s biomass composition in these two cases, as we do not have evidence about a different composition in these particular compounds for these two lineages. In the case of Rv1525, the fact that clinical isolates have these genes deleted suggests that there might be other genes compensating for the deletion [48].

We then deleted the genes affected by potentially deleterious SNPs that were prevalent within each lineage. As before, we checked if the removal of any of these genes blocked growth (see methods). Any of the models, besides L4 (the one that keeps all genes), were able to produce biomass. The number of genes with non-synonymous potentially deleterious SNPs that, when removed, blocked growth was: one for L1, seven for L2, four for L3, fourteen for L5, eighteen for L6, fifteen for L7, six for L8, fifteen for L9, eight for A1, seven for A2, twenty-nine for A3 and ten for A4. Again, we decided to keep all of them in the respective models. The description of what these genes code for and their PROVEAN score is included in Supplementary File S1. The curated lineage-specific GEMs are available in Supplementary File S2. The proportion of genomes per lineage sharing all genomic features that led to gene removal in each GEM was 100% for A1, 91.25% for A2, 87.25% for A3, 92.75% for A4, 99.44% for L1, 94.38% for L2, 84.27% for L3, 100% for L4, 93.82% for L5, 93.82% for L6, 96.07% for L7, 100% for L8 and 100% for L9. Therefore, our models are a good representation of the enzymatic gene content of each lineage’s isolates. FBA with the curated models yielded similar specific growth rate for all of them: between 0.35 and 0.37 h^−1^, except for L7, which had a specific growth rate of 0.21 h^−1^. This biomass production was considered sufficient for this lineage, as it grows at approximately half the speed of other *M. tuberculosis* lineages [54]. The models had approximately the same minimum medium requirements for sustaining the growth rate of L7 (Table 1). An exception was L7, which needed an additional 1 mmol/(gDW·h) of citrate and also consumed H^+^.

### 2.4. Analysis of Sampled Fluxes Shows Differences in Cholesterol Degradation, Central Carbon Metabolism and Mycolic Acids and Mycobactin Biosynthesis

**Figure 4:**
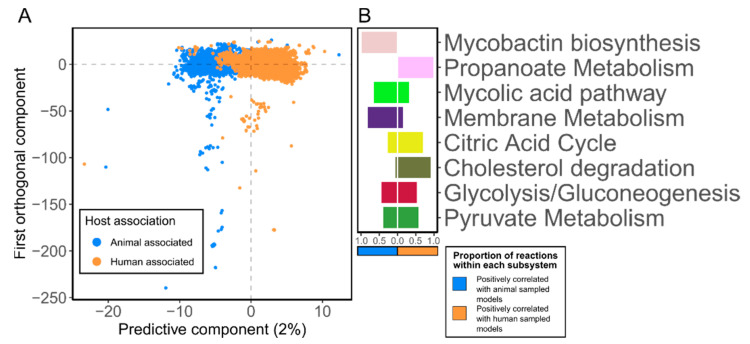
Comparative fluxomics between sampled flux distributions of genome-scale metabolic models of human- and animal-associated lineages. (**A**). Score plot of OPLS-DA (Orthogonal Partial Least Square Discriminant Analysis). The predictive component separates samples of animal-associated models from samples of human-associated models. (**B**). Proportion of reactions fluxes within each one of the altered subsystems positively correlated either to human or animal association (determined by the sign of the loading value of predictive component of each reaction, positive means correlated to human, negative to animal).

We could not see any separation between animal- and human-associated FBA flux distributions, neither with Principal Component Analysis (Supplementary Figure S3) nor with a random forest model. As the differences in metabolic fluxes between models of either animal- or human-associated models might manifest in conditions other than maximum growth, we decided to sample the solution space of each model. We obtained 1000 samples for each model, simulating Middlebrock 7H9 OADC medium supplemented with cholesterol. Cholesterol is an important nutrient within the macrophages and we wanted to explore possible associations between fluxes in cholesterol pathways and host preference [55,56]. PCA was not able to clearly separate samples by lineage or by host association (Supplementary Figure S4), despite the fact that lineages occupied distinct areas in the bidimensional space. However, fitting an orthogonal partial least squares discriminant analysis (OPLS-DA) [57], which is a supervised approach, showed a significant correlation between sampled fluxes and host association (R^2^ = 0.778, Q^2^ = 0.774, *p-*value = 0.01, Accuracy = 0.98) (Figure 4A). Using OPLS-DA’s variable importance in projection (VIP) values, we determined 385 differential reactions (VIP ≥ 1.0) (Supplementary Table S5). The differences in fluxes of these reactions across samples contributed significantly to the sample separation by host association. Finally, we used these reactions to perform an over-representation analysis (ORA). OPLS-DA model loadings for the predictive component were used to determine the contribution of each reaction in the perturbed subsystems for the separation by host association (Supplementary Figure S5 and Table S4). We found eight subsystems significantly altered between human- and animal-associated models (Table 2). The subsystem with the lowest *p*-value was cholesterol degradation. Other altered subsystems are related to carbon metabolism: glycolysis/gluconeogenesis, citric acid cycle, pyruvate metabolism and propanoate metabolism. Additionally, we also found altered subsystems related to cell wall and membrane processes such as mycolic acid pathway and membrane metabolism. Finally, mycobactin biosynthesis was also perturbed.

Glycolysis/gluconeogenesis and pyruvate metabolism have a similar number of reaction fluxes positively correlated with animal-associated models and with human-associated models, indicating differential flux diverting between human- and animal-associated models (Figure 4B). However, most reaction fluxes within cholesterol degradation are positively correlated to human-associated sampled models. The same is observed for citric acid cycle and propanoate metabolism. On the other hand, all the reaction fluxes within mycobactin biosynthesis are higher in animal-associated sampled models. Membrane metabolism and mycolic acid pathway have more reaction fluxes correlated to animal models than to human ones (Figure 4B).

### 2.5. The Reactions Removed from the Models Are Not Correlated to Host Association

**Figure 5:**
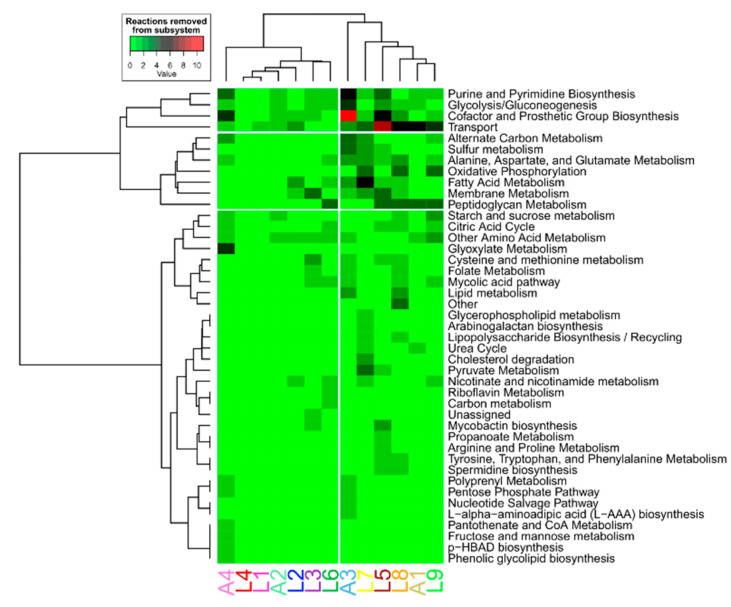
Hierarchical clustering analysis of the number of reactions removed of each subsystem within each lineage. Each row represents the number of reactions within each subsystem that were shut down when the genes were removed from the models, whereas the columns are the lineage-specific models. The clustering was performed with Euclidean and Ward D aggregation method.

One reaction may be catalyzed by the product of multiple genes, and one gene product may catalyze multiple reactions. As such, gene absence does not necessarily imply reaction absence. We therefore investigated if flux differences in the altered subsystems were caused by similar numbers of absent reactions in each subsystem among lineages associated with the same host. A hierarchical clustering analysis was performed on the number of reactions removed within each subsystem per lineage (Figure 5, Supplementary Table S3). The subsystems with more removed reactions were transport, glycolysis/gluconeogenesis, purine and pyrimidine biosynthesis and cofactor and prosthetic group biosynthesis. The lineages did not aggregate according to host association: two main clusters were formed, both consisting of a mix of animal- and human-associated lineages. Therefore, the differences of fluxes observed in the OPLS-DA analysis should be caused by perturbations in the metabolic network introduced by mutations. These mutations affect distinct parts of the metabolic network but propagate through it and produce convergent fluxomic phenotypes correlated with host association.

### 2.6. The solution Space of iEK1011 2.0 and Derived Models Is Biased to Low Growth-Rate

**Figure 6:**
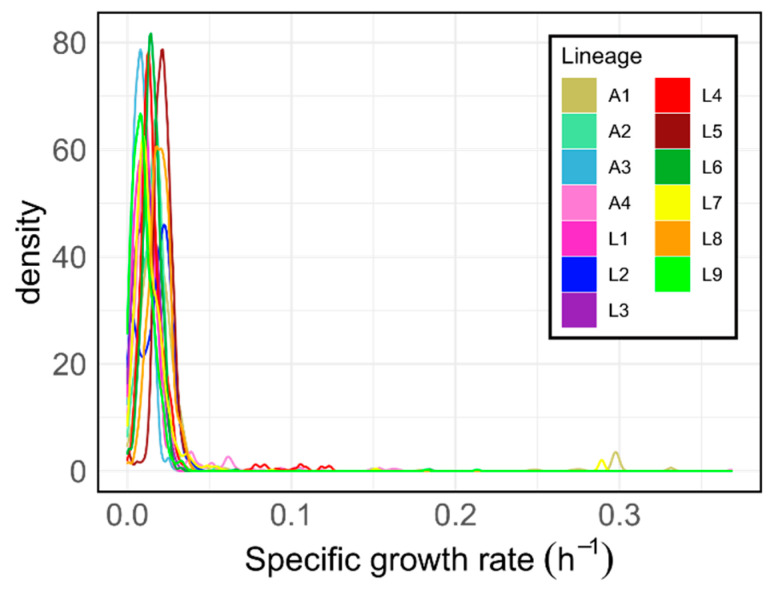
Density plots of biomass fluxes of the samples of each one of the lineage-specific genome-scale metabolic models. The solution space of each one of the models was sampled 1000 times in conditions mimicking Middlebrock 7H9 OADC + cholesterol and densities were obtained for each model.

We analyzed the distribution of biomass reaction flux across the sampled solutions of each model to assess the typical growth rate when the differences in fluxes are correlated to host association. We observed that at least 95% of the sampled flux distributions have a specific growth rate in the range of 0–0.05 h^−1^ for all the models (Figure 6). The models, as assessed by FBA, can support a much higher growth rate in simulated 7H9 medium supplemented with OADC and cholesterol (~0.37 h^−1^). It is in these low growth conditions where the differences between animal- and human-associated model fluxomes are observed. These differences should manifest in conditions where the bacteria are growing slowly. We compared the fluxes of exchange reactions between sampled and FBA solutions for each lineage’s model and found similar values (Supplementary Figure S6). With FBA all models were importing the maximum allowed flux of citrate, glucose, glutamate and glycerol (1 mmol/(gDW·h)), whereas for octadecenoate the import was 0.7 mmol/(gDW·h) when the maximum allowed is 1. The only model that imports cholesterol in FBA is L7. So, in FBA the models are consuming most of the carbon resources available, except for cholesterol. The fact that the import fluxes are similar in sampled solutions and in FBA indicates that the former are not in a starvation-like situation: they could grow at a higher rate with the carbon they are importing. This indicates that in the sampled solutions the carbon resources are being diverted to reactions other than biomass production. The import fluxes that are more different between the sampled solutions and FBA distributions are H_2_O and H^+^ (EX_h2o_e and EX_h_e). For all the models, sampled solutions import more H_2_O than the flux distributions obtained with FBA, except for L4 and L9 models. Regarding H^+^, import flux is always higher in FBA (Supplementary Figure S6).

## 3. Discussion

Human- and animal-associated strains of *M. tuberculosis* are highly similar at genomic level, and very few differences between lineages associated with different hosts could be found using both supervised and unsupervised methods. We corroborated that only two regions are lost in animal-associated lineages compared with humans: RD5 and RD1 [39]. RD5 has been deleted multiple times in animal-associated lineages [8,58,59], suggesting a possible involvement in host association. This region of difference includes three phospholipase C and two PPE genes that have been linked to virulence, nutrient obtention and antigen exposure [37,38,39,40]. The other genomic region which is absent from animal-associated lineages compared with human-associated ones is RD1, which has been deleted multiple times in animal clades: in A1 and A2 [8]. RD1 is the main modification involved in the attenuation of BCG vaccine strain [35,36,60,61]. SNPs distribution between animal- and human-associated strains show a similar pattern to what is observed for deletions, with few common SNPs to all animal-associated lineages but absent from all human-associated lineages. One of the SNPs markers found was the N88S substitution in the *iniA* gene, present in all the members of A2, A3 and A4 (Figure 1). The product of this gene is an efflux pump involved in the tolerance to isoniazid and ethambutol [33]. Another gene with potentially deleterious SNPs that affects only animal-associated lineages is *hemB.* All the members of A1, A3 and A4 are affected by different potentially deleterious SNPs in this gene. HemB protein participates in an early step of the synthesis of cobalamin and mycobactins, among other compounds [34,62,63,64]. Cobalamin is a cofactor of many enzymes and regulates gene expression [34]. Mycobactins are small molecules that sequester iron ions from ferritin, transferrin, lactoferrin and hemoglobin, the major sources of iron within the host’s cells [64,65,66]. A known mechanism of innate immune response for impairing bacterial proliferation consists of reducing iron availability [67]. *M. tuberculosis* has evolved to produce molecules with high affinity to iron, overcoming this limitation [68]. The SNPs in porphobilinogen synthase could be related to host adaptations, obtaining this nutrient from different host-specific sources. Finally, unsupervised analysis of the deletion data and the potentially deleterious SNPs shows a phylogeny-like pattern, where animal lineages form a monophyletic cluster with L5, L6 and L9 [8]. The identified genomic signatures might be partially involved in host association, but they are not sufficient to explain host specificity.

Although random forest models can detect gene to gene interaction [69], small-effect interactions that happen between genes that are distant in the metabolic network might not be detected. Additionally, different combinations of genomic features, apparently unrelated in the genome or in the metabolic network, could result in convergent metabolic phenotypes that correlate to host association. In this study, we integrated the genomic signatures characteristic to each one of *M. tuberculosis* lineages into a GEM, iEK1011 2.0, to build lineage-specific adaptations of it. We sampled the solution space of each one of the GEMs to assess if there were any flux differences correlated with host association without assuming the maximum growth rate. We determined the significantly perturbed metabolic subsystems between animal- and host-associated lineages. These convergent perturbations are not directly caused by deleted reactions within those subsystems, as the number of absent reactions within each subsystem was not correlated with host association. This indicates that host-correlated fluxes result from the convergent effect of removed reactions propagated through the metabolic network. These propagated effects produce flux distributions that allow for an accurate classification of each model’s host association.

The vast majority of the samples from all the models grew significantly slower than the maximum growth rate estimated by FBA. As host-correlated fluxes were observed in sampled solutions and not in FBA flux distributions, the differences between reaction fluxes of animal- and human-associated models are observed in a situation of low growth-rate. This slow growth was not caused by a lower carbon uptake. We hypothesize that the low growth-rate dependent correlation between the fluxome and host association might reflect host-specific adaptations to stress or differences in dormancy state. The altered subsystems were related with lipid/carbon metabolism and to cell wall/membrane processes, except for mycobactin biosynthesis. Cholesterol, whose degradation is the most significantly altered and is positively correlated to human association, is an important carbon source during macrophage infection [55,56]. Another lipid related subsystem significantly altered was the mycolic acid pathway. Mycolic acids are important in evading host immune response and have been shown to be upregulated in stress conditions [70,71]. Central carbon metabolism has also been shown to be altered in stress conditions. In particular, glycolysis, citrate cycle, and pyruvate metabolism, which we found altered in our models, have been shown to be impacted in oxidative and acidic stress and in starvation conditions [25]. Finally, as we mentioned before, mycobactins mediate the obtention of iron in depleted environments such as the phagosome [64,65,66]. The SNPs detected in *hemB* gene were not finally included in the models, as this gene is necessary for the gain of biomass flux. 

Altered subsystems such as mycolic acid pathways have products that are included in the model’s biomass composition. However, we did not alter it during the adaptation of iEK1011 2.0. Biomass composition differences in mycolic acids have been described in L1, L2, L4 and L6 [72], however we lack information about other compounds and other lineages. The flux differences in these pathways suggest that differences in cell wall components are likely to exist between animal- and human-associated lineages.

The process of building lineage-specific metabolic models has some limitations. The reference for the mapping of the sequencing reads is a reconstructed ancestral sequence based on the genomic content of a reference strain (H37Rv). [73]. Therefore, we are lacking the few genes present in other strains that might be absent from H37Rv genome. Furthermore, the base GEM we used to build the models is also based in H37Rv. We are therefore only accounting for genes that are absent in each one of the lineages compared with H37Rv, but we are not adding any reaction that might exist in lineages different than L4. This limitation led us to detect genes that are essential in H37Rv but appear deleted in some lineages: Rv1525 (*wbbL2*) in L1 and Rv3281 (*accE5*) in L8, both involved in the synthesis of cell wall components. These lineages must have other genes not present in H37Rv that are compensating for the deletions. The annotation of the complete genomes available of these lineages does not account for genes with the same functional category we report as missing [6,41]. Because an important proportion of *M. tuberculosis* genes is not annotated (NC_000962.3), it is likely that the genes filling the detected metabolic gaps remain unknown. Our approach has been proven useful in the detection of such gaps. However, we might not be seeing the full array of metabolic differences between the lineages due to the absence of genes in our reference. However, the lack of complete genomes of some *M. tuberculosis* lineages, including almost all animal lineage and the incompleteness of its annotation, hampers the task of including these genes in our models. Nevertheless, in a species such as *M. tuberculosis*, where the genomic identity is higher than 99% [14], the number of genes which are absent in H37Rv but present in other lineages might be very small. Therefore, the impact of not including them in our GEMs should not be important, if their presence is not necessary for bacterial viability.

Regarding the impact of SNPs, premature stop codons are likely affecting protein function but assessing the functional effect of missense SNPs is difficult. In this study we used PROVEAN, one of the best performing sequence homology-based tools [31,74]. However, still, among the genes affected by non-synonymous SNPs predicted to be deleterious we needed to keep some of them in order to have functional models. Inaccuracies in prediction of such impact could explain this. Other explanation could be the existence of other genes in the lineage carrying the missing function, or that promiscuous enzymes included in the model can catalyze a reaction that is not considered in the model. Alternatively, the function could be impaired but not totally suppressed. Furthermore, some of the genes with predicted deleterious SNPs were non-essential in the experimental datasets of H37Rv but essential in the model, indicating model inaccuracies.

As the fluxomic differences we observed happen in a growth range expected in situations where the bacterium is stressed or in dormancy state, the model could be expanded to integrate processes within the host. Previous attempts to build models reproducing host-pathogen dynamics have been useful to study how the metabolic network is rewired in response to antimycobacterial drugs [27]. This integration could serve to analyze if there are drug-induced responses correlated to host association. Another layer of complexity would be adding gene expression data to further constrain reaction flux values. Altogether, the workflow used in this study might serve as a platform for the study of other complex phenotypes related in some way to metabolism in other microorganisms.

## 4. Materials and Methods

### 4.1. Computational Workflow Overview

**Figure 7:**
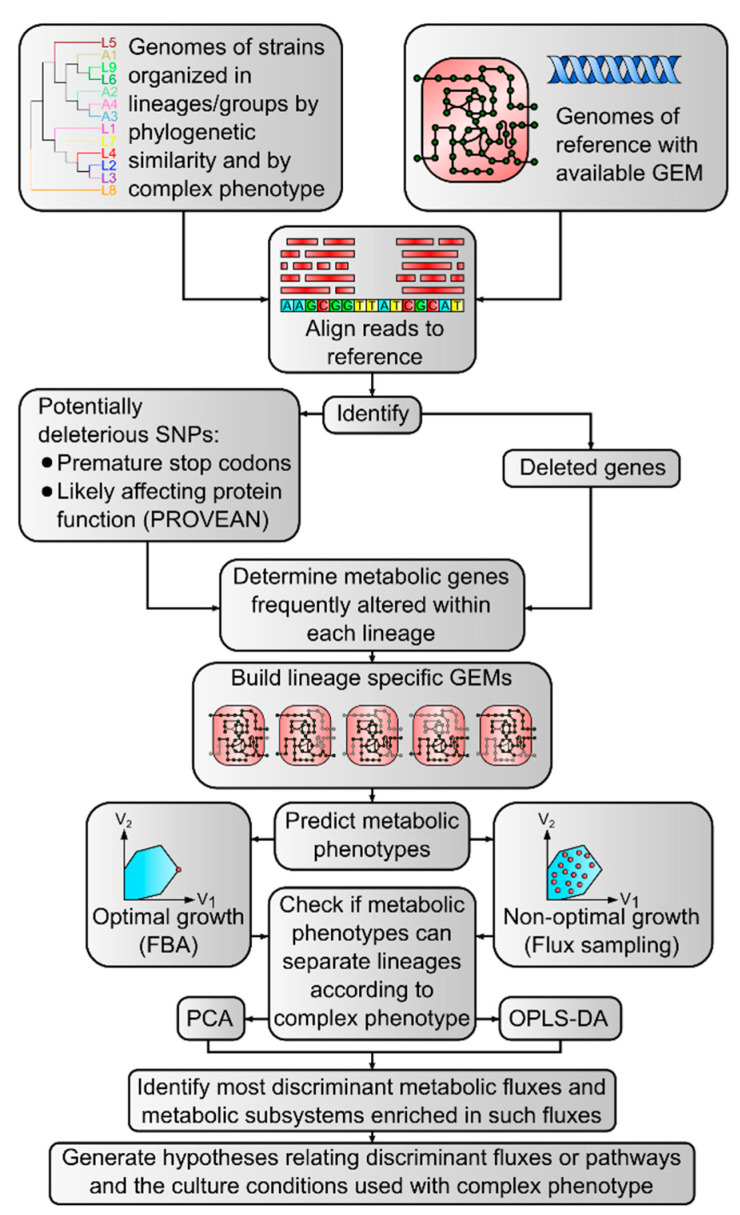
Workflow used in the analysis. We started with genomic sequences of *Mycobacterium tuberculosis* that aggregate in lineages and exhibit a complex phenotype (host association), and a reference sequence with a genome-scale metabolic model (GEM) available. Short reads mapping to reference served to identify deletions and potentially deleterious SNPs. We identified which of them were prevalent within each lineage and adapted the reference GEM to build lineage-specific versions. The resulting models were used to predict metabolic phenotypes, to later check if they can be used to separate lineages according to the complex phenotype (host association in our case). By identifying the most discriminant fluxes and the enriched subsystems in these fluxes, we can generate hypotheses relating the discriminant fluxes and the complex phenotype.

We designed a computational workflow (Figure 7) to predict metabolic phenotypes for collections of bacterial strains, using their genomic sequences to adapt a reference GEM. The main aim was to generate explanatory hypotheses connecting genomic variants to complex phenotypes through their impact in the organism’s metabolic network.

The input data for the workflow are a collection of genomes organized in groups. Each group should contain phylogenetically related strains with a common phenotype of interest, which in our case was the host association of the lineage. One of the groups should contain a reference strain with an available GEM. All genomes are compared with the reference to identify SNPs and deleted regions. SNPs are filtered to keep only the ones with potential deleterious effects. Within each group of related strains, metabolic genes that are frequently deleted or targets of deleterious SNPs are identified. The high frequency of alterations within the group suggests that it is a conserved event with functional consequences. The metabolic reactions associated with these frequently altered genes are blocked in the reference GEM to originate a model specific for that group of strains. Group-specific GEMs are tested for their ability to generate biomass in appropriate culture media. Some of the blocked reactions can be essential for growth, which suggests that the strains lacking the corresponding genes should have alternative genes not present in the reference genome to catalyze such reactions. In those situations, the list of blocked reactions is redefined, keeping intact the minimum number of reactions affected by potentially deleterious polymorphisms to allow the model to generate a steady-state flux distribution with biomass formation. The set of group-specific viable GEMs is used to predict metabolic phenotypes under various conditions: using different culture media, optimizing growth-rate, or sampling the solution space of each model. The aim of these variations is to find conditions where the predicted metabolic phenotypes (the steady-state metabolic fluxes) discriminate the groups of strains according to the complex phenotypes under study. The trial-and-error search for these discriminating conditions is guided by the analysis of unsupervised and supervised learning models applied to the generated metabolic flux distributions. Once the discriminating conditions are found, the supervised learning models are analyzed to extract the individual metabolic fluxes that have an important contribution to the accuracy of phenotype classification. These most important fluxes and the associated pathways can suggest new hypotheses explaining how the complex phenotype of interest can be influenced by changes in metabolic network activity. In the remaining sections of this section, we detail the methods used to apply this workflow to discover potential metabolic network determinants of host association in *M. tuberculosis* strains.

### 4.2. Genomic Data from Clinical Isolates

The accession numbers of the Illumina sequences analyzed in this study (*n* = 26,972), the lineage each isolate belongs to and the details about the sequencing run are included in the Supplementary Table S1.

### 4.3. Mapping and Variant Calling

Mapping and variant calling was performed as in Coscollá et al. 2021 [7]. Briefly, the FASTQ were trimmed with Trimmomatic v 0.33 (SLIDINGWINDOW 5:20) to remove Illumina adaptors and low quality reads [75], excluding from downstream analysis the ones shorter than 20 bp. Overlapping paired-end reads of 15 nucleotides size were merged with SeqPrep v1.2 (https://github.com/jstjohn/SeqPrep, accessed on 23 June 2020). The resultant reads were aligned to the reconstructed ancestral sequence of *M. tuberculosis* obtained by Comas et al. [73] using BWA v 0.7.13 (mem algorithm) [76]. Duplicated reads were marked by the Mark Duplicates module of Picard v 2.9.1 (https://github.com/broadinstitute/picard, accessed on 23 June 2020) and excluded. Reads with an alignment score corresponding to more than 7 mismatches per 100 bp were excluded using Pysam v 0.9.0 to avoid false positives (https://github.com/pysam-developers/pysam, accessed on 23 June 2020). SNPs were called with SAMtools v 1.2 mpileup [77] and VarScan v 2.4.1 [78]. Only SNPs that reached fixation within an isolate were considered (within-host frequency, i.e., SNP frequency within the reads of the same sample, higher 90%), calling the ancestor state otherwise. Mixed infections or contaminations were discarded: genomes with more than 1000 variable positions with within-host frequencies between 90 and 10% and genomes for which the number of within-host SNPs was higher than the number of fixed SNPs (SNPs with within-host frequency higher than 90%) were excluded. Additionally, genomes with mean read depth <15× (after all the referred filtering steps) were excluded too. All SNPs were annotated using snpEff v4.11 [79], in accordance with the *M. tuberculosis* H37Rv reference annotation (NC_000962.3). SNPs within regions such as PPE and PE-PGRS, phages, insertion sequences and in regions with at least 50 bp identities to other regions in the genome were excluded from the analysis, as in the paper by Stucki et al. [80]. Customized scripts were used to calculate mean coverages per gene corrected by the size of the gene. Gene deletions were determined as regions of the reference genome without read coverage.

### 4.4. Determination of Potentially Deletereous SNPs

The total set of SNPs observed in all the isolates was filtered to keep only SNPs that produced a premature stop codon. In parallel we kept non-synonymous SNPs and used PROVEAN to predict if the substitution will have a deleterious effect (score ≤ −2.5) [31]. The potentially deleterious SNP dataset consisted of a mix of these two datasets. 178 and 400 genomes were randomly selected from each one of the human- or animal-associated lineages, respectively, with sample replacement in the cases where the sample size is higher than the number of available sequences in the lineage. In this way, a representative sample of each lineage was achieved, and class imbalance was avoided. We determined the presence or absence of each potentially deleterious SNP within each genome. These data constituted the starting point for downstream SNPs analysis. 

### 4.5. Unsupervised and Supervised Analyses of Potentially Deleterious SNPs

For the unsupervised analysis we first counted how many times each gene was affected by potentially deleterious SNP within a lineage and obtained the average per lineage. A correspondence analysis was then performed using FactoMineR R package. For the supervised analysis we fitted a random forest model to the sampled genomes matrix obtained in 4.4. We used the caret R package for this purpose [81]. Accuracy was assessed with 10-fold cross validation. 

### 4.6. Unsupervised Analysis of the Deletion Data

Principal Component Analysis was performed with R’s base function on deletion percentage of each one of H37Rv ORFs. Results were plotted with factoextra R package. The PCAs of enzymatic genes and of genes included in iEK1011 GEM were performed analogously, previously filtering the ORFs to keep the ones that had either an entry in KEGG with an Enzyme Commission (EC) number assigned or the ones that appear in iEK1011 2.0 model. Correspondence Analysis was carried out with FactoMineR, with previous binarization of deletion data with a 15% threshold (gene is deleted if 15% or more of the sequence is lost). The input data were the counts of isolates within each lineage carrying deletions on each one of the genes included in iEK1011 2.0 model.

### 4.7. Random Forest of Deletion Data

We used the caret R package [81] for random forest analysis of lineage-specific deletions, determined by the deletion percentage of each one of the clinical isolates. A sampling of the isolates was performed to avoid class imbalance and lineage underrepresentation (lineages like L2 and L4 are overrepresented, whereas others such as L8, L9 and A1 are underrepresented). For this we computed the rounded median of the number of isolates per human lineage to determine the number of sequences sampled from each human-associated lineage. We multiplied this number by the total number of human-associated isolates and divided it by the total number of animal-associated isolates to obtain the number of isolates to sample from each animal-associated lineage. If the number of samples was bigger than the number of sequences available for the lineage, sampling was carried out with replacement. This sampling was carried out in each one of the 10 rounds of cross validation. 

### 4.8. Lineage-Specific Model Construction and Curation

We used COBRApy for obtaining all the GEMs, curate them and to carry out the simulations [82]. The lineage-specific GEMs were adapted from iEK1011 2.0, an improved version of the iEK1011 model [26,30]. This model is a representation of the H37Rv strain, which belongs to L4; therefore, the intact model was considered to be the L4 model. To build the lineage-specific models, the deletions and the potentially deleterious SNP characteristics of each lineage were included in each model. Deletion data were binarized with a threshold of 85% of coverage in the reference. A chi-squared test was used to obtain differentially deleted genes between each lineage and L4 (Benjamini-Hochberg adjusted *p*-value ≤ 0.05). The lists of differentially deleted genes were filtered to keep just the ones that are deleted in more than the 85% of the target lineage. This constituted the first list of genes to remove per lineage model. To these lists the SNPs annotated as stop gain codon and the missense SNPs predicted to be deleterious by PROVEAN that were present in more than the 85% of each lineage were added. We then removed the reactions associated with these genes and simulated the resulting models maximizing growth rate in media conditions simulating Middlebrock 7H9 supplemented with OADC [30]. None of the models grew at this point; therefore, we found the minimum combination of removed genes that needed to be kept to obtain a growth rate comparable with the observed for the L4 model. When there were different options, i.e., different sets of genes with the same size that restore growth when kept, the genes kept were the ones with a higher PROVEAN score. Once we determined the genes that need to be removed from iEK1011 2.0 to obtain a lineage-specific functional model, we assessed how representative the model was for the whole lineage. For that, we determined the proportion of the genomes we sampled in 4.4 within each lineage that had either deletions or potentially deleterious SNPs in all the genes we removed for generating each GEM. 

### 4.9. Model Media Composition

The model’s medium settings for simulating Middlebrock 7H9 supplemented with OADC medium were obtained from López-Agudelo et al. [30]. The maximum allowed uptake flux for the compounds in the medium were: EX_glu__L_e (L-glutamate) = 1, EX_cu2_e (Cu_2_) = 1000, EX_btn_e (biotin) = 1, EX_pydxn_e (pyridoxine) = 1, EX_ca2_e (Ca_2_) = 1000, EX_mg2_e (Mg_2_) = 1000, EX_h_e (H^+^) = 1000, EX_k_e (K^+^) = 1000, EX_nh4_e (NH_4_^+^) = 10, EX_h2o_e (H_2_O) = 1000, EX_pi_e (phosphate) = 1, EX_cl_e (Cl^-^) = 1000, EX_o2_e (O_2_) = 20, EX_na1_e (Na^+^) = 1000, EX_so4_e (SO_4_^2−^) = 1000, EX_cit_e (citrate) = 1, EX_fe3_e = 5, EX_glyc_e (glycerol) = 1, EX_glc__D_e (D-glucose) = 1, EX_ocdca_e (octadecanoate) = 1. Flux units are mmol/(gDW·h). For the simulations with cholesterol, a maximum import flux of 1 mmol/(gDW·h) was allowed. 

### 4.10. Model Simulations

FBA was performed setting “BIOMASS__2” reaction as objective, simulating growth in Middlebrock 7H9 supplemented with OADC. PCA of the obtained flux distributions was carried out with R 3.6. Flux sampling was carried out with optGpSampler method within COBRApy (N = 1000) [83]. The medium was 7H9 supplemented with OADC and cholesterol (1 mmol/gDW/h). The medium settings represent the maximum flux that can be imported into the system, but each value is not fixed. 

### 4.11. Sampled Flux OPLS-DA

OPLS-DA model of sampled flux distributions was carried out with ropls R package [84] aiming to predict the host association of the corresponding lineage. The number of orthogonal components was fixed to 3. R^2^ and Q^2^, key parameters for assessing the validity of the model, were assessed with 7-fold cross validation. The significance of the model was determined by permutation test (*n* = 100). The *p*-value corresponds to the proportion of Q^2^_perm_ above Q^2^. With a *p-*value below 0.05 we considered the model significant. The accuracy was computed by dividing the number of observations correctly classified by the model by the total number of observations. The loadings of the predictive component of the model were extracted to determine the correlation of each reaction flux to host association. Variable Importance in Projection (VIP) values were also obtained, to assess the contribution of each reaction flux to the classification. 

### 4.12. Subsystem Over-Representation Analysis

Differential reaction fluxes between samples of human- and anima-associated GEMs were determined with VIP values. VIP is defined as the weighted sum of squares of the PLS weight, reflecting the importance of the variable to the entire model. We considered reaction fluxes with VIP ≥ 1.0 as significant [25,85,86]. An over-representation analysis was used to determine metabolic subsystems enriched in reactions with differential fluxes between animal- and human-associated models, based on a one-sided Fisher exact test (adjusted by Benjamini-Hochberg method, significant with an adjusted *p*-value lower or equal to 0.05) [87]. The subsystems tested were the ones iEK1011 2.0 has assigned to each one of the reactions. 

### 4.13. Removed Reactions Hierarchical Clustering Analysis

The reactions that were blocked in each model, when the genes affected by potentially deleterious mutations were removed, were determined according to iEK1011 2.0 Gene Protein Reaction (GPR) rules. The reactions were grouped by subsystem and a count of how many reactions were lost in each subsystem per lineage model was obtained. A hierarchical clustering analysis was performed on this dataset, using Euclidean distance and Ward D aggregation method. The heatmap was obtained with gplots R package [88]. 

### 4.14. Biomass Density Plots

The densities of the biomass sampled fluxes for each one of the lineage-specific GEMs were obtained with the ggplot2 R package [89]. 

## Figures and Tables

**Table 1:** Minimum growth requirements of lineage-specific models for sustaining the growth rate of the L7 model. The unit is mmol/(gDW·h).

	Lineage	L1	L2	L3	L4	L5	L6	L7	L8	L9	A1	A2	A3	A4
Nutrient														
Citrate		0.00	0.00	0.00	0.00	0.00	0.00	1.00	0.00	0.00	0.00	0.00	0.00	0.00
Fe^3+^		0.00	0.00	0.00	0.00	0.00	0.00	0.00	0.00	0.00	0.00	0.00	0.00	0.00
D-Glucose		1.00	1.00	1.00	1.00	1.00	1.00	1.00	1.00	1.00	0.60	1.00	0.37	1.00
L-Glutamate		1.00	1.00	1.00	1.00	1.00	1.00	1.00	1.00	1.00	1.00	1.00	1.00	1.00
Glycerol		1.00	1.00	1.00	1.00	1.00	1.00	1.00	1.00	1.00	0.00	1.00	1.00	1.00
H^+^		0.00	0.00	0.00	0.00	0.00	0.00	11.02	0.00	0.00	0.00	0.00	0.00	0.00
NH_4_^+^		0.33	0.33	0.41	0.33	0.33	0.33	0.33	0.33	0.33	0.33	0.33	0.33	0.33
O_2_		10.90	10.90	10.93	10.90	10.90	11.41	10.56	11.39	10.90	12.63	10.90	12.20	10.90
octadecanoate		0.45	0.45	0.46	0.45	0.45	0.38	0.05	0.46	0.45	0.73	0.45	0.63	0.45
phosphate		0.22	0.22	0.22	0.22	0.22	0.22	0.22	0.22	0.22	0.22	0.22	0.22	0.22
SO_4_		0.03	0.03	0.03	0.03	0.03	0.03	0.03	0.03	0.03	0.03	0.03	0.03	0.03

**Table 2:** Altered subsystems between animal- and human-associated models. Each model’s flux space was sampled in conditions mimicking Middlebrock 7H9 OADC supplemented with cholesterol. The significantly differential reactions between models of animal- and human-associated lineages were determined with a multivariate analysis (OPLS-DA, VIP ≥ 1.0), and an over-representation analysis was carried out with a Fisher exact test (adjusted *p-*value < 0.05).

Subsystem	Adjusted *p*-Value
Cholesterol degradation	2.67 × 10^−9^
Glycolysis/Gluconeogenesis	6.90 × 10^−4^
Citric Acid Cycle	2.89 × 10^−3^
Pyruvate Metabolism	1.47 × 10^−2^
Mycolic acid pathway	2.07 × 10^−2^
Membrane Metabolism	2.56 × 10^−2^
Mycobactin biosynthesis	3.97 × 10^−2^
Propanoate Metabolism	3.97 × 10^−2^

## Data Availability

The Illumina sequences used in this study are available at NCBI, identified with the accession numbers indicated in Supplementary Table S1. The code used in this study is available at: https://gitlab.com/guisana/mapspipe, accessed on 5 December 2021; https://gitlab.com/guisana/delspipe, accessed on 5 December 2021; https://gitlab.com/guisana/snpspipe, accessed on 5 December 2021; https://gitlab.com/guisana/modspipe, accessed on 5 December 2021; https://github.com/guisantagui/linmodsbuild, accessed on 5 December 2021.

## Supplementary Materials

The following are available online at https://www.mdpi.com/article/10.3390/biom12030376/s1, Figure S1: Correspondence Analysis of all the potentially deleterious SNPs, Figure S2: Unsupervised analysis of the deletion data of the genes included in iEK1011 2.0 genome-scale metabolic model, Figure S3: Principal Component Analysis of FBA flux distributions of the lineage-specific genome-scale metabolic models, Figure S4: Principal Component Analysis of the sampled solution space of each one of the lineage-specific genome-scale metabolic models, Figure S5: OPLS-DA loadings for the significantly altered subsystems between animal- and human-associated sampled models, Figure S6: Difference of exchange fluxes between sampled models and FBA flux distribution of metabolites in Middlebrock 7H9 OADC + cholesterol for each lineage’s model, Table S1: Illumina genomes information, Table S2: iEK1011 2.0 reaction information, Table S3: Removed reactions per lineage model, Table S4: OPLS-DA variable coefficients, Table S5: Statistically significant reaction fluxes between samples of animal- and human-associated models, File S1: Description of the genes removed from iEK1011 2.0 to generate lineage-specific GEMs, File S2: Lineage-specific genome scale metabolic models.
