# Supplementary material for: In Silico Exploration of Mycobacterium tuberculosis Metabolic Networks Shows Host-Associated Convergent Fluxomic Phenotypes
Source: Biomolecules. 2022 Feb 28;12(3):376. doi: 10.3390/biom12030376 (PMC8945471; doi:10.3390/biom12030376)
Supplement: Supplementary file 1 [file biomolecules-12-00376-s001.zip › supplementary-Figures.zip!/Figure S1.pdf]

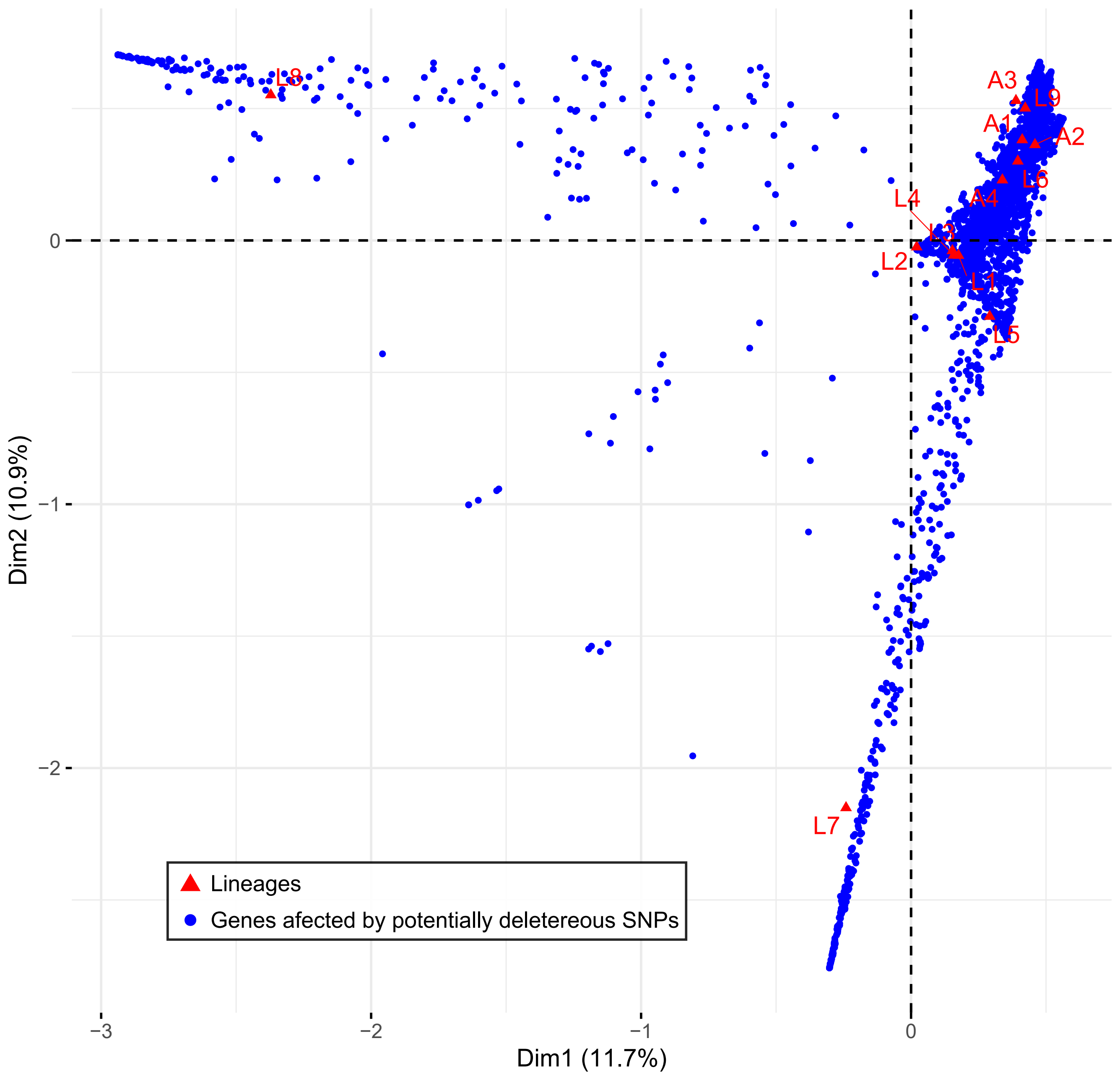

L8
A3
L9
A1
A2
L6
L4
A4
L3
0
L2
L1
L5
−1
−2
L7
Dim2 (10.9%)
Lineages
Genes afected by potentially deletereous SNPs
−3
−2
−1
0
Dim1 (11.7%)
