# Supplementary material for: In Silico Exploration of Mycobacterium tuberculosis Metabolic Networks Shows Host-Associated Convergent Fluxomic Phenotypes
Source: Biomolecules. 2022 Feb 28;12(3):376. doi: 10.3390/biom12030376 (PMC8945471; doi:10.3390/biom12030376)
Supplement: Supplementary file 1 [file biomolecules-12-00376-s001.zip › supplementary-Figures.zip!/Figure S2.pdf]

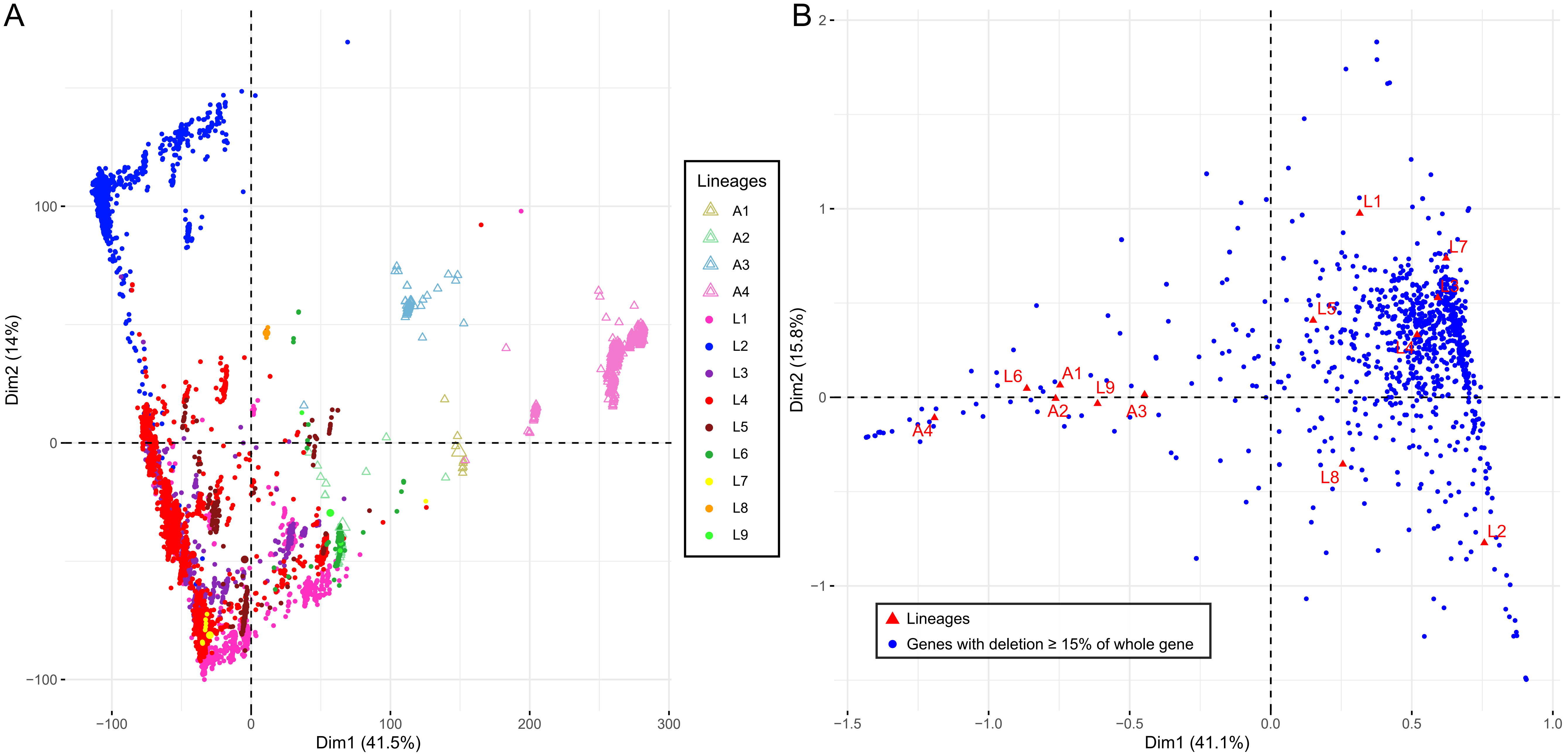

A
Dim2 (14%)
Dim1 (41.5%)
100
0
−100
−100
0
100
200
300
Lineages
A1
A2
A3
A4
L1
L2
L3
L4
L5
L6
L7
L8
L9
B
Dim2 (15.8%)
Dim1 (41.1%)
2
1
0
−1
−1.5
−1.0
−0.5
0.0
0.5
1.0
L1
L7
L3
L5
L4
L6
A1
L9
A2
A3
A4
L8
L2
Lineages
Genes with deletion ≥ 15% of whole gene
