# Supplementary material for: In Silico Exploration of Mycobacterium tuberculosis Metabolic Networks Shows Host-Associated Convergent Fluxomic Phenotypes
Source: Biomolecules. 2022 Feb 28;12(3):376. doi: 10.3390/biom12030376 (PMC8945471; doi:10.3390/biom12030376)
Supplement: Supplementary file 1 [file biomolecules-12-00376-s001.zip › supplementary-Figures.zip!/Figure S3.pdf]

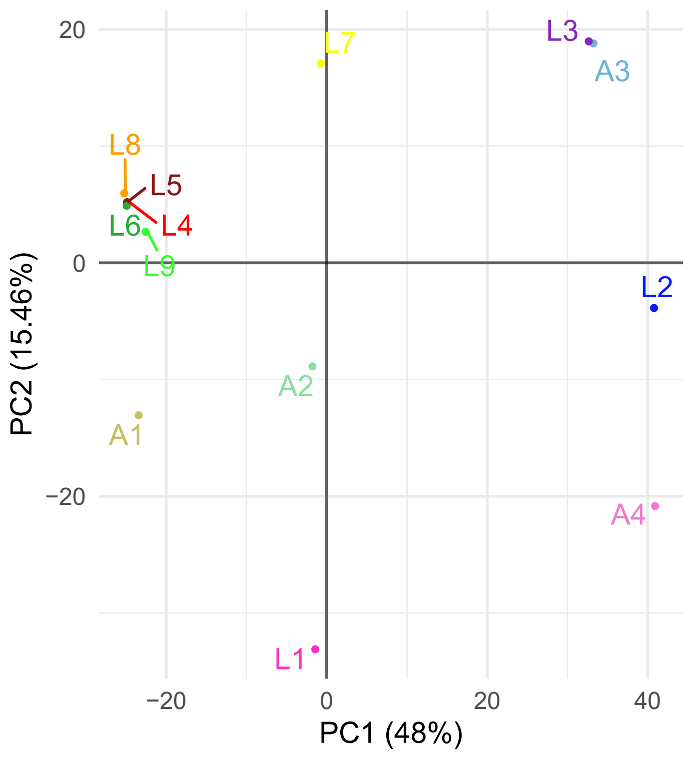

L3
A3
L7
L8
L5
L6
L4
L9
L2
A2
A1
A4
L1
20
0
−20
−20
0
20
40
PC1 (48%)
PC2 (15.46%)
