# Supplementary material for: In Silico Exploration of Mycobacterium tuberculosis Metabolic Networks Shows Host-Associated Convergent Fluxomic Phenotypes
Source: Biomolecules. 2022 Feb 28;12(3):376. doi: 10.3390/biom12030376 (PMC8945471; doi:10.3390/biom12030376)
Supplement: Supplementary file 1 [file biomolecules-12-00376-s001.zip › supplementary-Figures.zip!/Figure S4.pdf]

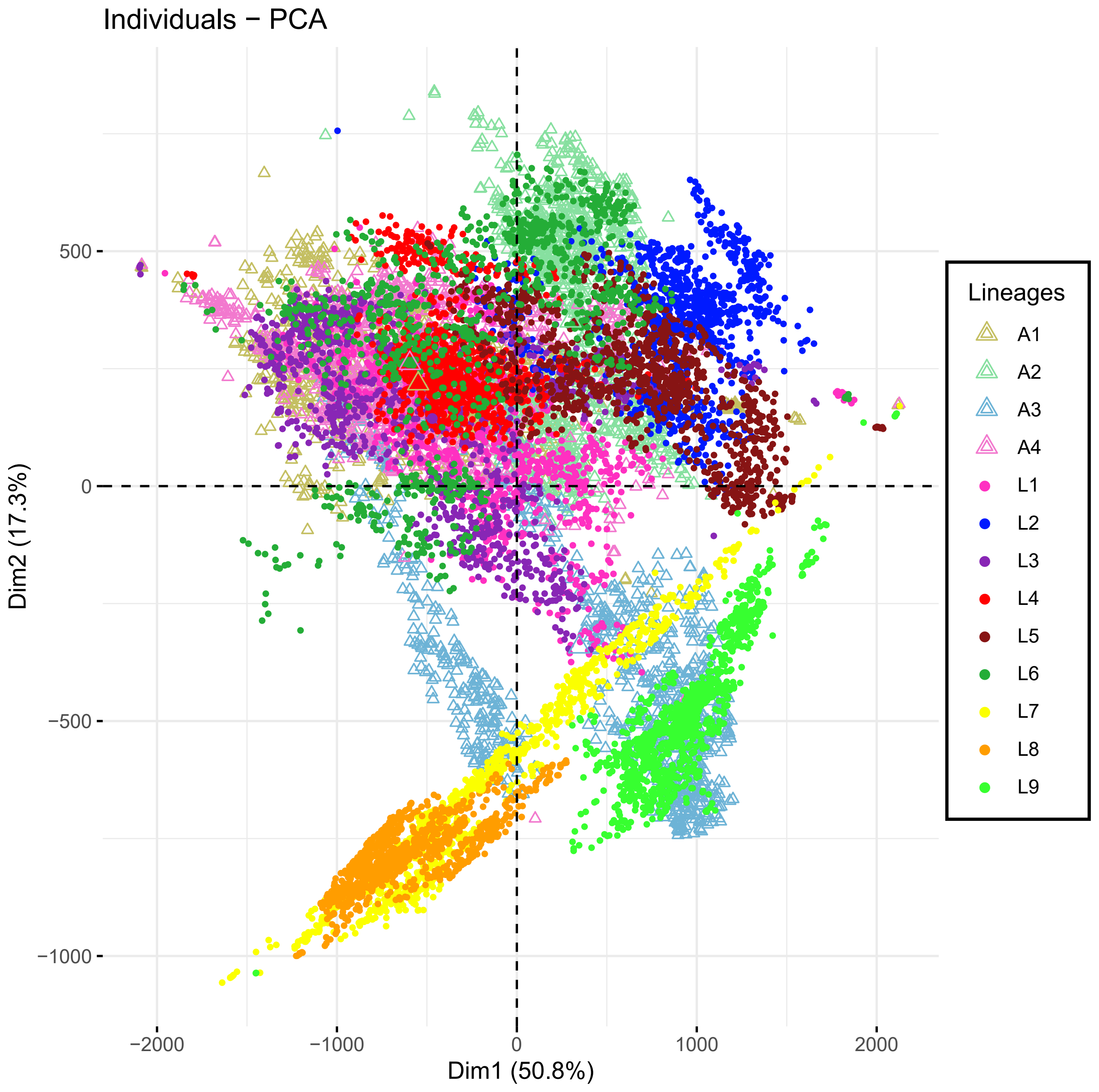

Individuals − PCA
Dim2 (17.3%)
Dim1 (50.8%)
500
0
−500
−1000
−2000
−1000
0
1000
2000
Lineages
A1
A2
A3
A4
L1
L2
L3
L4
L5
L6
L7
L8
L9
