# Supplementary material for: In Silico Exploration of Mycobacterium tuberculosis Metabolic Networks Shows Host-Associated Convergent Fluxomic Phenotypes
Source: Biomolecules. 2022 Feb 28;12(3):376. doi: 10.3390/biom12030376 (PMC8945471; doi:10.3390/biom12030376)
Supplement: Supplementary file 1 [file biomolecules-12-00376-s001.zip › supplementary-Figures.zip!/Figure S5.pdf]

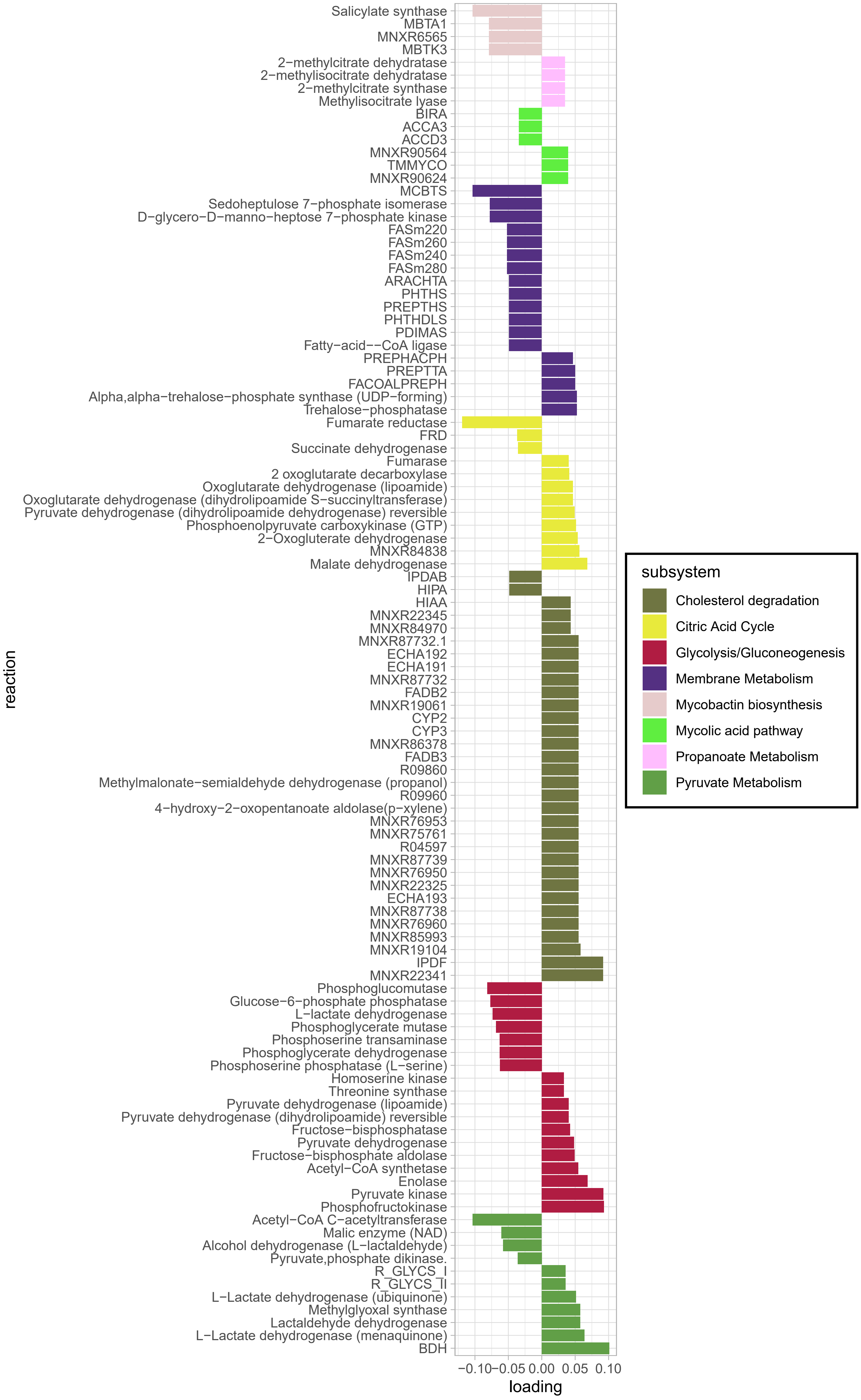
Salicylate synthase
MBTA1
MNXR6565
MBTK3
2−methylcitrate dehydratase
2−methylisocitrate dehydratase
2−methylcitrate synthase
Methylisocitrate lyase
BIRA
ACCA3
ACCD3
MNXR90564
TMMYCO
MNXR90624
MCBTS
Sedoheptulose 7−phosphate isomerase
D−glycero−D−manno−heptose 7−phosphate kinase
FASm220
FASm260
FASm240
FASm280
ARACHTA
PHTHS
PREPTHS
PHTHDLS
PDIMAS
Fatty−acid−−CoA ligase
PREPHACPH
PREPTTA
FACOALPREPH
Alpha,alpha−trehalose−phosphate synthase (UDP−forming)
Trehalose−phosphatase
Fumarate reductase
FRD
Succinate dehydrogenase
Fumarase
2 oxoglutarate decarboxylase
Oxoglutarate dehydrogenase (lipoamide)
Oxoglutarate dehydrogenase (dihydrolipoamide S−succinyltransferase)
Pyruvate dehydrogenase (dihydrolipoamide dehydrogenase) reversible
Phosphoenolpyruvate carboxykinase (GTP)
2−Oxogluterate dehydrogenase
MNXR84838
Malate dehydrogenase
IPDAB
HIPA
HIAA
MNXR22345
MNXR84970
MNXR87732.1
ECHA192
ECHA191
MNXR87732
FADB2
MNXR19061
CYP2
CYP3
MNXR86378
FADB3
R09860
Methylmalonate−semialdehyde dehydrogenase (propanol)
R09960
4−hydroxy−2−oxopentanoate aldolase(p−xylene)
MNXR76953
MNXR75761
R04597
MNXR87739
MNXR76950
MNXR22325
ECHA193
MNXR87738
MNXR76960
MNXR85993
MNXR19104
IPDF
MNXR22341
Phosphoglucomutase
Glucose−6−phosphate phosphatase
L−lactate dehydrogenase
Phosphoglycerate mutase
Phosphoserine transaminase
Phosphoglycerate dehydrogenase
Phosphoserine phosphatase (L−serine)
Homoserine kinase
Threonine synthase
Pyruvate dehydrogenase (lipoamide)
Pyruvate dehydrogenase (dihydrolipoamide) reversible
Fructose−bisphosphatase
Pyruvate dehydrogenase
Fructose−bisphosphate aldolase
Acetyl−CoA synthetase
Enolase
Pyruvate kinase
Phosphofructokinase
Acetyl−CoA C−acetyltransferase
Malic enzyme (NAD)
Alcohol dehydrogenase (L−lactaldehyde)
Pyruvate,phosphate dikinase.
R_GLYCS_I
R_GLYCS_II
L−Lactate dehydrogenase (ubiquinone)
Methylglyoxal synthase
Lactaldehyde dehydrogenase
L−Lactate dehydrogenase (menaquinone)
BDH
reaction
−0.10
−0.05
0.00
0.05
0.10
loading
subsystem
Cholesterol degradation
Citric Acid Cycle
Glycolysis/Gluconeogenesis
Membrane Metabolism
Mycobactin biosynthesis
Mycolic acid pathway
Propanoate Metabolism
Pyruvate Metabolism
