# Supplementary material for: In Silico Exploration of Mycobacterium tuberculosis Metabolic Networks Shows Host-Associated Convergent Fluxomic Phenotypes
Source: Biomolecules. 2022 Feb 28;12(3):376. doi: 10.3390/biom12030376 (PMC8945471; doi:10.3390/biom12030376)
Supplement: Supplementary file 1 [file biomolecules-12-00376-s001.zip › supplementary-Figures.zip!/Figure S6.pdf]

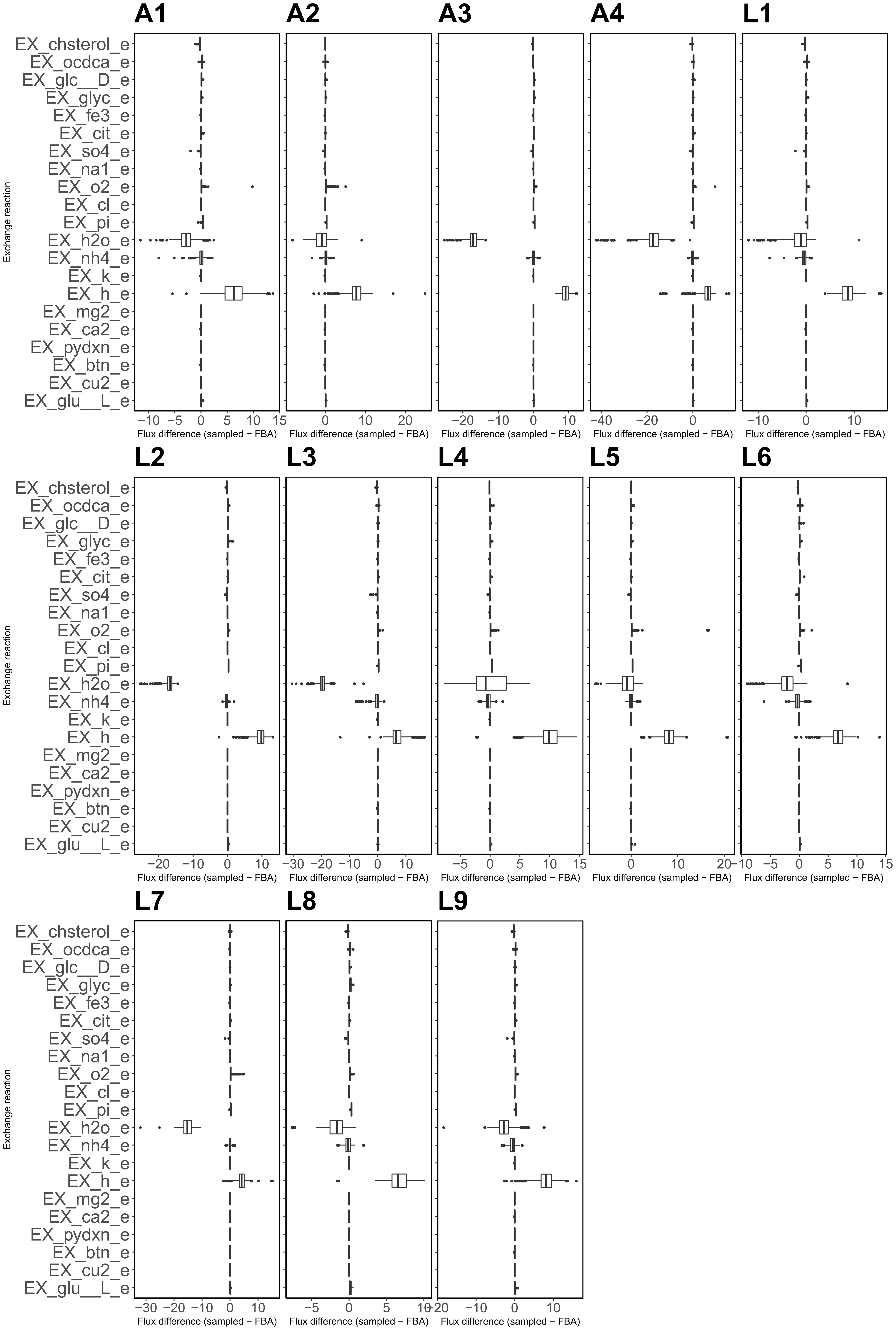
A1
A2
A3
A4
L1
L2
L3
L4
L5
L6
L7
L8
L9
Exchange reaction
EX_chsterol_e
EX_ocdca_e
EX_glc__D_e
EX_glyc_e
EX_fe3_e
EX_cit_e
EX_so4_e
EX_na1_e
EX_o2_e
EX_cl_e
EX_pi_e
EX_h2o_e
EX_nh4_e
EX_k_e
EX_h_e
EX_mg2_e
EX_ca2_e
EX_pydxn_e
EX_btn_e
EX_cu2_e
EX_glu__L_e
Flux difference (sampled − FBA)
